# NIH Roadmap for Medical Research

**Published:** 2008

**Authors:** Lori Wolfgang Kantor

**Affiliations:** *Science Editor for* Alcohol Research & Health

The National Institutes of Health (NIH) Roadmap for Medical Research is a collection of far-reaching initiatives designed to transform the Nation’s medical research capabilities and improve the translation of research into practice. As described below, the Roadmap consists of three major themes: new pathways to discovery, research teams of the future, and reengineering the clinical research enterprise.

## New Pathways to Discovery

The following initiatives within this theme aim to improve the understanding of complex biological systems and to develop tools that will advance biomedical research.

### Building Blocks, Biological Pathways, and Networks

Efforts are being made to develop new technologies to help researchers studying the biological pathways and networks that facilitate communication among genes, molecules, and cells. One of the central components of these networks is the set of proteins encoded by an organism’s genes (i.e., the proteome). Tools are being developed that will enable researchers to determine, in real time, the amounts, locations, physiological effects, and interactions of large numbers of individual proteins within a single cell.

Another focus of this initiative is to provide researchers with new analytical tools to better understand the metabolic components and networks within cells. For example, new technologies may help researchers measure local concentrations of carbohydrates, lipids, amino acids, and other metabolites within a single cell or even a specific part of a single cell. Specific areas of research emphasis include approaches that are addressing the widely fluctuating range of metabolite concentrations and complexity of metabolite mixtures, the vast number of unidentified compounds present within single samples, and the dynamic nature of the cell’s entire set of metabolites. This type of comprehensive information may pave the way for the development of better methods to detect metabolic differences between normal and diseased cells.

### Molecular Libraries and Imaging

Small molecules, often with molecular weights of 500 or below, are important for researchers studying molecular and cellular functions. Such molecules are valuable for treating diseases, and most medicines marketed today are from this class. With small-molecule libraries, biomedical researchers in the public sector can have access to the large-scale screening necessary to identify small molecules that can be used as chemical probes for genes, cells, and metabolic and biochemical pathways. This will lead to new ways to explore the functions of genes and signaling pathways in health and disease.

The imaging component of this initiative focuses on the imaging of molecules or molecular events in biological systems that span the scale from single cells to whole organisms. Ultimately, this effort may enable personalized profiles of cell and tissue function, which may lead to more individualized approaches to diagnosing and treating disease.

### Structural Biology

This effort aims to map the molecular shapes of proteins in the body. This involves the development of rapid, efficient, and dependable methods to produce protein samples that researchers can use to determine the three-dimensional structure of a protein. The new effort is catalyzing what currently is a time-consuming process into a streamlined routine, helping researchers clarify the role of protein shape in health and disease.

### Bioinformatics and Computational Biology

These initiatives are creating a national software engineering system to evaluate, combine, and visualize the large amounts of data collected through biomedical research. Through a computer-based grid, biologists, chemists, physicists, computer scientists, and physicians anywhere in the country will be able to share and analyze data using a common set of software tools.

### Nanomedicine

An offshoot of nanotechnology, nanomedicine refers to highly specific medical intervention at the molecular level for curing disease or repairing damaged tissues, such as bone, muscle, or nerve. The Nanomedicine Roadmap Initiative calls for the creation of Nanomedicine Development Centers, which are focusing on gathering information about the structures, processes, and networks inside cells. Researchers will use this information to develop tools to detect and correct biological defects in unhealthy cells.

### The Human Microbiome Project

Little is known about the communities of microbial cells that inhabit healthy human bodies. The Human Microbiome Project aims to study these cells and their role in human health and disease. New DNA sequencing technologies have created a field of research, called metagenomics, that allows the comprehensive study of microbial communities, even those composed of organisms that cannot be cultivated experimentally. Instead of examining the genome of an individual bacterial strain that has been grown in a laboratory, the metagenomic approach allows analysis of genetic material derived from complete microbial communities harvested from natural environments. In the human microbiome project, this method will complement genetic analyses of known isolated strains, providing unprecedented information about the complexity of human microbial communities.

### Epigenomics

Epigenetics involves the study of changes in the regulation of gene activity and expression that are not dependent on gene sequence. Epigenomics is the global analyses of epigenetic changes across the entire genome. The NIH Roadmap epigenomics program is based on the hypothesis that the origins of health and susceptibility to disease are, in part, the result of epigenetic regulation of the genetic blueprint. In particular, this hypothesis suggests that epigenetic mechanisms that control stem cell differentiation and organ formation contribute to the biological response to endogenous and exogenous forms of stimuli that result in disease. The epigenomics program aims to develop comprehensive reference epigenome maps and new technologies for comprehensive epigenomic analyses.

## Research Teams of the Future

The following initiatives within this theme are designed to encourage scientists and scientific institutions to test a variety of models for conducting research.

### High-Risk Research

The NIH Director’s Pioneer Award program is designed to support individual researchers with innovative approaches to major challenges in biomedical research.

### Interdisciplinary Research

A series of awards will be established to make it easier for scientists to conduct interdisciplinary research. Other initiatives are designed to change NIH policies and procedures. For example, rather than recognizing only a single Principal Investigator (PI) for every award, NIH is moving toward recognition of multiple PIs for any award.

### Public–Private Partnerships

Initiatives are promoting and facilitating partnerships among researchers in academia, the Government, and the private sector.

## Reengineering the Clinical Research Enterprise

The following initiatives within this theme are central to the goal of moving research results more quickly into clinical settings.

### Clinical Research Networks

This initiative is focused on improving and expanding existing clinical research data networks and standardizing data reporting to improve networking. Another goal is to determine the feasibility of a National Electronic Clinical Trials/ Research Network (NECTAR), which will provide the informatics infrastructure that will serve as the backbone for interconnected and interoperable research networks.

### Clinical Outcomes Assessment

This initiative aims to develop new technologies to improve the assessment of patient-reported clinical outcomes, such as fatigue, pain, and mood changes.

### Clinical Research Training

Efforts within this initiative are designed to expand and strengthen the clinical research workforce by supporting career development for clinical researchers, increasing the number of clinical researchers, diversifying the settings in which clinical research is conducted, and providing clinical research training for medical and dental students.

### Clinical Research Policy Analysis and Coordination

These initiatives address the difficulties clinical researchers confront in satisfying the multiple requirements of diverse regulatory and policy agencies. NIH is working to standardize reporting requirements and streamline policies.

### Translational Research

Initiatives in this group are designed to accelerate the translation of research findings to patient care, partly by fostering the development of a new discipline of clinical and translational science that will be broader and deeper than the current separate domains of translational research and clinical investigation.

See http://nihroadmap.nih.gov/ for more information on the NIH Roadmap initiatives.

